# Evidence for Combining Conservative Treatments for Adhesive Capsulitis

**DOI:** 10.31486/toj.23.0128

**Published:** 2024

**Authors:** Jordan L. Hill

**Affiliations:** Ochsner Therapy and Wellness, Driftwood Clinic, Ochsner Clinic Foundation, Kenner, LA

**Keywords:** *Bursitis*, *conservative treatment*, *frozen shoulder*, *range of motion–articular*, *shoulder pain*

## Abstract

**Background:** Adhesive capsulitis, also known as frozen shoulder, is a challenge to treat clinically. Common first-line treatment options are suprascapular nerve block (SSNB), intra-articular corticosteroid (IACS) injection, hydrodilatation, and physical therapy. This literature review summarizes each of these conservative treatments and discusses the evidence base for combining treatment options for potential additive benefits to improve patient outcomes (ie, pain, range of motion [ROM], and shoulder function).

**Methods:** The PubMed and Google Scholar databases were searched using the search terms “adhesive capsulitis,” “frozen shoulder,” “corticosteroids,” “physical therapy,” “suprascapular nerve block,” “hydrodilatation,” and “conservative care.” Pertinent articles were identified and synthesized to provide a comprehensive review of 4 common conservative treatments for adhesive capsulitis.

**Results:** Combining SSNB with physical therapy and/or IACS injection and combining IACS injection with physical therapy have support in the literature for improving shoulder pain, ROM, and function, while hydrodilatation and physical therapy seem to offer some additive benefits for improving shoulder ROM when used as adjunct treatments for adhesive capsulitis.

**Conclusion:** Adhesive capsulitis remains a challenge to treat clinically with much still unknown regarding treatment optimization. For the foreseeable future, first-line conservative management will continue to be the mainstay of managing adhesive capsulitis. Thus, knowing how to best use and optimize these various options—both individually and in combination—is vital for effective treatment.

## INTRODUCTION

Adhesive capsulitis, also known as frozen shoulder, is a common musculoskeletal disorder characterized by pain (including night pain), progressive loss of active and passive shoulder range of motion (ROM), and joint mobility restrictions that overall lead to markedly impaired shoulder function.^1^ In primary, or idiopathic, adhesive capsulitis, an underlying etiology or associated condition cannot be identified.^2^ Primary adhesive capsulitis affects approximately 2% to 5% of the general population, and the incidence increases in patients with diabetes and thyroid disease.^3-5^ In secondary adhesive capsulitis, an underlying etiology or associated condition can be identified.^2^ While several detailed clinical staging models exist,^6-8^ the clinical course of adhesive capsulitis is generally marked by a painful, inflammatory phase that eventually evolves into a fibrotic phase; treatment has historically addressed the primary clinical feature of each phase.^9^

While surgical treatments for adhesive capsulitis, including arthroscopic capsular release and manipulation under anesthesia, have support in the literature, surgery is typically reserved for refractory cases.^10^ First-line treatment centers on conservative management. Numerous nonsurgical adhesive capsulitis treatment options exist, but 4 of the most widely practiced are suprascapular nerve block (SSNB), intra-articular corticosteroid (IACS) injection, hydrodilatation, and physical therapy. Each option, to various degrees, has support in the literature as a stand-alone treatment for adhesive capsulitis.^11^ This review explores how these 4 commonly used nonsurgical treatment options compare. Additionally, because combining treatments is in line with common clinical practice for managing adhesive capsulitis,^12^ this literature review explores whether combining 1 or more of these 4 treatment options has potential additive benefits for improving patient outcomes (ie, pain, ROM, and shoulder function).

## METHODS

The PubMed and Google Scholar databases were searched using the search terms “adhesive capsulitis,” “frozen shoulder,” “corticosteroids,” “physical therapy,” “suprascapular nerve block,” “hydrodilatation,” and “conservative care.” Pertinent articles were identified and synthesized to provide a comprehensive review of 4 common conservative treatments for adhesive capsulitis.

## CONSERVATIVE TREATMENTS FOR ADHESIVE CAPSULITIS

### Suprascapular Nerve Block

SSNB is a viable treatment option for patients with either acute or chronic nonspecific shoulder pain.^13,14^ A hallmark symptom of adhesive capsulitis is pain. As an intervention, SSNB can provide pain relief by targeting the suprascapular nerve that provides up to 70% of the sensory innervation to the glenohumeral joint.^15^ The procedure involves the injection of a local anesthetic (commonly bupivacaine or ropivacaine), with or without ultrasound guidance, via either a posterior or anterior approach. A major advantage of SSNB is that it can be performed as a same-day, office-based procedure, and physical therapy can be started immediately postprocedure.

For pain alleviation, SSNB has been shown to have significant benefit vs placebo in the short term for treating adhesive capsulitis.^16,17^ When comparing the effectiveness of SSNB and IACS injection for pain, ROM, and function, some studies reported that SSNB was inferior to IACS injection,^16^ while others showed comparable efficacy or, in some instances, concluded that SSNB led to better pain relief and function in both the short and long terms.^14,18,19^ In a meta-analysis by Chang et al, SSNB was deemed more effective than physical therapy for pain relief, with the effects lasting up to 12 weeks.^16^ However, the inclusion criteria for the Chang et al study allowed various types of shoulder pain lasting >1 month, and adhesive capsulitis was only one of those types.

Also pertinent to this review are the results of studies examining the effects of combining SSNB with other commonly used treatment modalities for adhesive capsulitis, such as physical therapy and IACS injection. In a 2015 study by Klç et al, an SSNB given prior to the initiation of a physical therapy program, which included ROM, stretching, strengthening exercises, and electrotherapy, improved the pain and function of patients with adhesive capsulitis compared to patients who received physical therapy alone.^20^ Jung et al, in a 2019 retrospective cohort study, concluded that an SSNB significantly added to the treatment efficacy of an IACS injection for pain and function in patients with adhesive capsulitis.^21^ These treatment benefits were maintained for a minimum of 1 year postintervention. However, a more recent study (2021) by Gencer Atalay et al that compared both the short- and long-term effects of using an SSNB as an adjunct to IACS injection demonstrated a positive benefit only in the immediate (first hour) time point postintervention.^22^ No additional benefits for pain, ROM, function, or quality of life were observed past the 3-week time point. The authors acknowledged the disparity between their study findings and those of others,^14,19,21^ citing the fact that previous studies used a mixture of local anesthetic and corticosteroid as an injectant for the SSNB intervention, while a local anesthetic injection alone (bupivacaine) was used in their study to administer the SSNB. Despite the heterogeny of these findings, practitioners have advocated using SSNB in combination with other treatment modalities to provide added treatment benefit for patients with adhesive capsulitis.^19,21^

### Intra-Articular Corticosteroid Injection

An IACS injection is the injection of a corticosteroid directly into the glenohumeral joint, often in combination with a local anesthetic. Based on what is currently understood about adhesive capsulitis pathophysiology,^23,24^ the recommendation is to administer IACS injection during the early stage when the prominent clinical features are pain and high joint irritability.^12,25,26^ Pathophysiology studies of adhesive capsulitis, such as the work by Hettrich et al,^27^ support this general administration time frame, as IACS injection has been shown to decrease the amount of transforming growth factor-β1, fibroblasts, and myofibroblasts, all of which have been linked to the pathologic capsular tissue changes in adhesive capsulitis. Two systematic reviews, a meta-analysis, and a Cochrane review support the benefits of IACS injection for improving pain and function in patients with adhesive capsulitis in the short term (up to 6 weeks) compared to placebo.^25,28-30^ These benefits diminish, however, in the medium to long term (>12 weeks to 24 weeks). In their meta-analysis, Wang et al reported that IACS injection resulted in increased shoulder flexion, abduction, and external rotation ROM in both the short and medium terms.^28^

Different aspects of the IACS injection procedure have been explored in the literature within the context of treating adhesive capsulitis,^12^ including single vs multiple injections,^31^ type of corticosteroid,^32^ imaging guidance vs no imaging guidance,^33^ injection site (intra-articular vs subacromial vs rotator interval),^34,35^ and dose optimization.^36^

While the research acknowledges the positive benefits of IACS injection as a stand-alone treatment for adhesive capsulitis, evidence also supports the potential additive effects of combining IACS injection with other treatment modalities. In a randomized controlled trial, Carette et al compared the efficacy of a single IACS injection, a supervised physical therapy program, a combination of IACS injection and supervised physical therapy, and placebo in patients with adhesive capsulitis.^37^ A single IACS injection was more effective than both the placebo and supervised physical therapy in improving shoulder pain and function at the 6-week time point, while the combination of IACS injection and supervised physical therapy led to faster restoration of shoulder ROM compared to the other 3 treatment groups. A prospective study by Anjum et al in 2020 also reported improved pain and greater recovery of shoulder ROM with the combination of IACS injection and a physical therapy program.^38^ The 2020 systematic review (65 studies) and meta-analysis (34 studies) by Challoumas et al found IACS injection to be the most beneficial nonoperative treatment intervention for adhesive capsulitis compared to physical therapy or subacromial corticosteroid injections, with benefits lasting as long as 6 months.^39^ In addition, Challoumas et al reported that the midterm benefits of IACS injection could be supplemented by adding hydrodilatation, physical therapy, and/or a simple home exercise program.^39^

### Hydrodilatation

The first documented study investigating hydrodilatation was published more than 50 years ago.^40^ However, compared to SSNB and IACS injection, hydrodilatation (also known as brisement or hydrodistension) is a lesser known and used treatment for adhesive capsulitis. In addition to pain, the other hallmark symptom of adhesive capsulitis is joint stiffness that leads to the loss of active and passive shoulder ROM in multiple planes of motion. Joint stiffness is usually observed in the mid to later stages of the adhesive capsulitis clinical course. Hydrodilatation, as the name implies, is injection of fluid into the glenohumeral joint with the goal of distending the joint,^10^ disrupting capsular fibrosis,^12^ and thus improving ROM. This intervention is an office-based procedure; patients are first screened for contraindications, informed of the risks and benefits, and educated on the postprocedure course.^41^ As with SSNB, patients undergoing hydrodilatation can start physical therapy the same day postprocedure.

A 2008 Cochrane review of hydrodilatation found silver-level evidence for hydrodilatation vs placebo for pain; ROM; and dysfunction at 3-, 6-, and 12-week follow-ups.^42^ Additional systematic reviews (2015 and 2021) further corroborated the efficacy of hydrodilatation for pain and functional improvement in patients with adhesive capsulitis compared to placebo.^43,44^ The systematic review and network meta-analysis by Zhang et al evaluated 32 nonsurgical interventions and identified hydrodilatation as the highest-ranking intervention for pain relief based on surface under the cumulative ranking curve values.^44^

Various aspects of the hydrodilatation procedure have been explored within the context of treating adhesive capsulitis: whether capsular rupture is necessary,^40,45^ single vs multiple hydrodilatations,^46^ air vs saline filler,^47^ and optimal injection volume.^48^ These investigations are less contentious than those comparing hydrodilatation to IACS injection.

A 2018 meta-analysis by Saltychev et al of 7 studies comparing hydrodilatation to IACS injection deemed hydrodilatation to have a small effect size and a number needed to treat of 12 for pain reduction and ROM improvement, suggesting low clinical significance for this procedure.^49^ However, in a larger meta-analysis performed in the same year, Lin and colleagues reported equivalent efficacy of hydrodilatation and IACS injection for pain reduction and functional improvement at 3 follow-up time points.^50^ Lin et al also reported that hydrodilatation yielded better external rotation ROM in the short to medium terms compared to IACS injection, leading them to recommend hydrodilatation as an early intervention for patients with predominant external rotation ROM limitations. A prospective study by Yoon et al compared IACS injection, subacromial injection, and hydrodilatation for pain reduction and increased ROM at 1-, 3-, and 6-month time points.^51^ Each group also received physical therapy, nonsteroidal anti-inflammatory drugs, and muscle relaxants. While hydrodilatation showed better results at the 1- and 3-month time points than IACS injection, these benefits could have resulted from the combination therapy rather than the hydrodilatation procedure alone; thus, the study design was flawed.

Several studies report that the addition of hydrodilatation to IACS injection can significantly improve shoulder ROM.^52-54^ These studies also included supervised physical therapy or a home exercise program as a part of their treatment regimen. Of the groups assessed in a randomized controlled trial by Park et al, the group receiving hydrodilatation, IACS injection, and intensive physical therapy showed the greatest improvement in pain, ROM, and function at the 4-week time point, leading the authors to recommend the combination of hydrodilatation, IACS injection, and physical therapy as the most effective treatment option for subacute adhesive capsulitis.^54^ In the Park et al study, *intensive physical therapy* referred to the use of various joint mobilization techniques, including passive accessory glenohumeral joint glides and active mobilizations with movement. The authors did not provide additional information regarding the dosage or intensity (mobilization grade) of the joint mobilization techniques used in their study.^54^

### Physical Therapy

Unlike SSNB, IACS injection, and hydrodilatation, which are each single interventions, physical therapy is a spectrum of interventions that includes manual therapy (such as joint mobilizations), therapeutic exercise (for strengthening and ROM), and modalities (ultrasound and electrical stimulation, functional dry needling, and neuromuscular reeducation). Because of the primary clinical manifestations of adhesive capsulitis, physical therapy is a mainstay of conservative management for this condition. Also, because of its wide scope, physical therapy can be used to treat adhesive capsulitis at any point along its continuum.^26,41^ In the early stages of adhesive capsulitis when pain dominates, physical therapy typically focuses on decreasing joint irritability. As pain decreases and joint stiffness increases in the mid to later stages of adhesive capsulitis, stretching and joint mobilization techniques are the physical therapy treatments of choice.^55,56^

In the 2020 systematic review and meta-analysis by Challoumas et al, physical therapy was found to improve outcomes compared with no treatment or placebo.^39^ As with the IACS injection and hydrodilatation procedures, various aspects of physical therapy have been researched within the context of treating adhesive capsulitis: different treatment regimens,^41,55,56^ joint mobilizations,^57,58^ stretching intensity,^55,56,59,60^ and specific interventions.^61,62^

Despite the regular use of physical therapy to treat adhesive capsulitis, its effectiveness has been challenged in the literature. Some reviews and studies have reported that physical therapy is not as effective as other commonly used adhesive capsulitis treatment options, such as IACS injection, when compared directly.^30,39,63,64^ In addition, the large heterogeny in physical therapy interventions^65^ and the variability of their application are challenges to conducting high-quality research and drawing firm conclusions about the effectiveness of physical therapy as a stand-alone treatment.^66^ Finally, a long-running narrative, originating from the seminal studies on adhesive capsulitis pathology^7,67,68^ and staging,^6,7^ promotes *supervised neglect* rather than physical therapy.^41^ Supervised neglect assumes that adhesive capsulitis is a completely self-resolving pathology, meaning that given enough time, the condition will resolve on its own. Work by Wong and colleagues refutes this long-running narrative and advocates for improved diagnostic testing and early treatment initiation—including physical therapy—for adhesive capsulitis.^69^

Physical therapy is typically employed as a part of a combined intervention approach based on treatment algorithms that match joint irritability.^41,56,70^ In the lone clinical practice guideline for adhesive capsulitis published by the Orthopaedic Section of the American Physical Therapy Association, the treatment with the highest recommendation is IACS injection with physical therapy (specifically joint mobilizations and stretching) used as an adjunct.^56^ The additive benefit of physical therapy combined with IACS injection has been established in the literature,^37-39,71^ and additional studies recommend hydrodilatation with supplementary physical therapy as an effective treatment option for adhesive capsulitis.^54,72^ One single-center study demonstrated improvements in shoulder ROM and patient-reported outcome measures (Shoulder Pain and Disability Index and Quick Disabilities of Arm, Shoulder, and Hand questionnaire) at 6 weeks to 6 months.^72^

## DISCUSSION

Adhesive capsulitis remains a challenge to treat clinically with much still unknown regarding treatment optimization. For the foreseeable future, first-line conservative management will continue to be the mainstay of managing adhesive capsulitis. Thus, knowing how to best use and optimize these various options—both individually and in combination—is vital for effective treatment.

SSNB shows good potential for pain control in the initial painful stage of adhesive capsulitis, and combining SSNB with physical therapy and/or IACS injection seems to have positive additive benefits on pain and shoulder function. The positive benefits of IACS injection seem to diminish after 6 to 12 weeks; however, moderate-level evidence supports combining IACS injection with physical therapy (or at least a home exercise program) for faster and greater improvement in shoulder ROM. Hydrodilatation shows potential as both a stand-alone and combination procedure for managing the stiffness aspect of adhesive capsulitis, especially for patients with a predominant external rotation ROM limitation. Hydrodilatation seems to offer the most benefits when combined with physical therapy and IACS injection. And while physical therapy does not seem to hold up well when compared directly to IACS injection, various aspects of physical therapy consistently provide additive benefit as an adjunct treatment for improving shoulder ROM and function in the short to medium terms for adhesive capsulitis.

## CONCLUSION

Combining SSNB with physical therapy and/or IACS injection and combining IACS injection with physical therapy each have support in the literature for improving shoulder pain, ROM, and function, while hydrodilatation and physical therapy seem to offer some additive benefits to improving shoulder ROM when used as adjunct adhesive capsulitis treatments.

## Notes

*This article meets the Accreditation Council for Graduate Medical Education and the American Board of Medical Specialties Maintenance of Certification competencies for Patient Care and Medical Knowledge.*
